# Critical appraisal of systematic reviews and meta-analyses: a step-by-step guide for nephrologists

**DOI:** 10.1080/0886022X.2025.2476736

**Published:** 2025-03-26

**Authors:** Wisit Cheungpasitporn, Wannasit Wathanavasin, Charat Thongprayoon, Wisit Kaewput, Mihály Tapolyai, Tibor Fülöp

**Affiliations:** ^a^ Division of Nephrology, Department of Medicine, Mayo Clinic, Rochester, MN, USA; ^b^ Nephrology Unit, Department of Medicine, Charoenkrung Pracharak Hospital, Bangkok Metropolitan Administration, Bangkok, Thailand; ^c^ Department of Military and Community Medicine, Phramongkutklao College of Medicine, Bangkok, Thailand; ^d^ Department of Nephrology, Szent Margit Kórház, Budapest, Hungary; ^e^ Medicine Service, Ralph H. Johnson VA Medical Center, Charleston, SC, USA; ^f^ Division of Nephrology, Department of Medicine, Medical University of South Carolina, Charleston, SC, USA

**Keywords:** Systematic reviews, meta-analyses, heterogeneity, risk of bias, nephrology, medical education

## Abstract

**Background:**

Systematic reviews and meta-analyses play a pivotal role in evidence-based medicine, including nephrology, by consolidating findings from multiple studies. To maximize their utility, rigorous quality assessment during peer review is essential. Challenges such as heterogeneity, bias, and methodological flaws often undermine these studies, necessitating a structured appraisal process.

**Methods:**

This guide outlines a framework for nephrologists on appraising systematic reviews and meta-analyses. Key areas include heterogeneity assessment using the I^2^ statistic, interpretation of forest plots for pooled effect estimates, and the use of funnel plots with Egger’s test to identify potential publication bias. Risk of bias is evaluated using RoB 2 for randomized controlled trials and ROBINS-I for non-randomized studies. Subgroup and sensitivity analyses, along with meta-regression, address heterogeneity and examine the robustness of findings.

**Results:**

The I^2^ statistic quantifies heterogeneity by estimating the proportion of variability in a meta-analysis. Funnel plots and Egger’s test help detect publication bias. Major biases, such as selection, performance, detection, and publication bias, are identified using structured tools like AMSTAR 2, Cochrane RoB 2, and ROBINS-I. The GRADE framework further assesses the overall certainty of the evidence. Emphasis is placed on PRISMA compliance, protocol pre-registration, and transparent reporting of statistical analyses, subgroup, and sensitivity assessments. The inclusion of grey literature remains optional.

**Conclusion:**

By focusing on key areas such as heterogeneity, risk of bias, and robust statistical methods, this guide enables nephrologists to critically appraise systematic reviews and meta-analyses, fostering better clinical decision-making and improved patient care in nephrology.

## Introduction

As nephrologists stepping into the role of peer reviewers, we play a crucial role in maintaining the quality and integrity of published research. With the significant surge in medical information, systematic reviews and meta-analyses are essential to evidence-based nephrology, addressing the inconsistencies and conflicts among different research findings. This type of research often builds upon previous meta-analyses; therefore, assessing when the last review on the topic was published and identifying how the current study advances beyond it is critical. Such advancements could include updated evidence, improved methodological approaches, or addressing previously unexplored aspects of the question.

These studies are increasingly utilized to guide decision-making in healthcare and public policy [1,2]. When executed appropriately, systematic reviews and meta-analyses are regarded as strong evidence, ranking at the highest level in the hierarchy of evidence [3]. To ensure the value of a systematic review, the updated PRISMA 2020 statement [4] provides revised reporting guidelines that incorporate recent advancements in the methods used to identify, select, evaluate, and synthesize studies. Evidence regarding sources of bias in systematic reviews has accumulated, leading to the development of new tools [5,6] for evaluating the conduct of these reviews. Additionally, the terminology used to describe particular review processes has also changed, with the transition from assessing ‘quality’ to assessing the ‘certainty’ of the body of evidence. This guide will help us navigate the critical appraisal process of these studies and provide insights into tools and programs that can streamline the process, ensuring efficiency and transparency.

The landscape of nephrology research is rapidly evolving, with an increasing number of systematic reviews and meta-analyses being published each year [7,8]. These studies synthesize data from multiple primary research sources, offering a comprehensive overview of current evidence on various nephrological topics. However, the quality of these reviews can vary significantly, potentially impacting clinical decision-making. As peer reviewers, our task is to critically evaluate the methodological rigor, statistical analyses, and overall validity of these studies. This guide aims to equip us with the necessary skills and knowledge to effectively assess systematic reviews and meta-analyses, ultimately contributing to the advancement of evidence-based practice in nephrology.

## Understanding systematic reviews and meta-analyses

- Systematic Review (SR) is a structured, comprehensive search and review of relevant literature based on a clearly defined research question, employing predefined eligibility criteria (inclusion and exclusion of individual studies) and methods to select studies. Critical appraisal of individual studies and evaluating the certainty of evidence across studies are essential components of SR. These features distinguish SRs from other narrative reviews and enable them to provide a more objective summary of evidence for specific clinical question.
- Meta-Analysis (MA) is a statistical technique that combines results from multiple studies addressing the same question to provide a pooled effect estimate across larger population. The objectives are to identify whether an effect is present and to determine if it is positive or negative, ideally culminating a single summary estimate of the effect. The results of a meta-analysis can enhance the precision of effect estimates, address questions not considered in the individual studies, resolve controversies from seemingly conflicting findings, and generate new hypotheses. However, not all systematic reviews include a meta-analysis, as not all topics are suitable or feasible for data pooling.
- Network Meta-Analysis is an advanced statistical method that allows the comparison of multiple interventions across different studies, even if those interventions have not been directly compared in head-to-head trials. This is accomplished by integrating both direct and indirect evidence. Direct evidence comes from randomized controlled trials (RCTs), while indirect evidence is gathered through one or more common comparators. The combination of these types of evidence is known as mixed evidence.
- Diagnostic Test Accuracy (DTA) Meta-Analysis is a specialized form of meta-analysis that evaluates the performance of diagnostic tests by pooling sensitivity, specificity, and other metrics across multiple studies. Systematic reviews and meta-analyses are widely used in nephrology to evaluate the efficacy of interventions, risk factors for disease progression, and diagnostic test performance. Diagnostic test accuracy (DTA) meta-analyses play a pivotal role in assessing the reliability of biomarkers, imaging modalities, and laboratory tests commonly used in nephrology. For example, meta-analyses evaluating serum cystatin C as a marker of kidney function, urinary neutrophil gelatinase-associated lipocalin (NGAL) for predicting acute kidney injury (AKI), or the role of artificial intelligence in kidney pathology highlight the importance of structured evaluation of diagnostic tests in nephrology research.

## Key components to evaluate: step-by-step guide to appraising systematic reviews and meta-analyses (Table 1)

### Step 1: evaluation of research question and protocol

The initial step entails assessing the research question and protocol, which is crucial for reviewers to grasp the study’s focus. A well-executed systematic review or meta-analysis should feature a clearly defined research question that is recognizable to the reader and typically follows the PICO framework (population, intervention, comparison/control, and outcome) [10]. A well-formulated research question should strike a balance between being too broad and too narrow. A research question that is overly broad may face criticism for the risk of ‘mixing apples and oranges’ (heterogeneity), which can compromise its validity. Conversely, if the question is too narrow, it may limit the generalizability of the findings to other contexts and populations [18]. Following the evaluation of the research question, it is essential to check if the researcher has registered the protocol in an international database, such as the PROSPERO registry [9], the International platform of registered systematic review and meta-analysis protocols (INPLASY) [19] or the Open science framework (OSF) [20]. This step is crucial as it indicates that the researcher is addressing key aspects related to ensuring transparency, preventing duplication, and potentially minimizing reporting bias.

In nephrology, well-formulated research questions are particularly crucial due to the complexity of kidney disease phenotypes and the heterogeneity of patient populations. For example, systematic reviews investigating interventions for diabetic kidney disease must clearly define whether the focus is on early-stage CKD or end-stage kidney disease (ESKD), as treatment effects may vary. Additionally, given the evolving landscape of nephrology research, protocol registration in databases like PROSPERO is essential to minimize bias and duplication, particularly in rapidly expanding areas such as artificial intelligence applications in kidney disease.

### Step 2: evaluation of literature search

To ensure a comprehensive and exhaustive literature search for relevant primary studies, multiple databases (more than two), such as MEDLINE, CENTRAL, and Embase, should be systematically searched to decrease the risk of missing relevant studies [21]. In accordance with PRISMA guidelines [4,11], the final manuscript should include full search strategies that appropriately detail the search terms used, with at least one database presented in the main manuscript; the others can be provided in the supplementary material. When conducting a search on PubMed, it is strongly recommended to include MeSH terms. These terms are organized hierarchically, with more specific terms listed under broader categories. Generally, a detailed list of search terms was consistently used for each component of the PICO framework. This information should be presented in a main text table or as supplementary data in a table or appendix. Of note, at least two reviewers should conduct the literature search independently with the reviewers’ initials specifically stated in the manuscript. Any discrepancies in the final selection of articles to be included should be addressed and resolved through consensus.

To ensure a comprehensive and up-to-date search, it is essential that the literature search be as recent as possible. Ideally, the search should not be older than six months from the time of manuscript submission. If it has been longer, the authors should consider updating the search and re-running the data synthesis to incorporate any new studies that may have emerged. This approach enhances the reliability of the review findings, particularly in fast-evolving fields like nephrology, where new research is published frequently.

Since the objective of a systematic review is to summarize all available evidence on a topic, it is recommended to include studies without restrictions related to language, publication year, or other factors during the search process [22]. However, due to limited time or financial resources, this may not always be possible. For example, if there is a justified reason for language restriction (e.g. due to time constraints in rapid reviews), they should only be applied during the study selection stage, not during the literature search. Studies excluded due to language should be listed in the PRISMA flow diagram, along with the reason for exclusion, to ensure transparency regarding the number of eligible reports in other languages. This approach allows future reviewers to consider these studies when examining previous SRs on the same topic. Similarly, effectively implementing date range restrictions in SRs requires a clear rationale for determining the timeframe, whether is to capture recent evidence, manage workload, or ensure relevance. The research question should guide this decision, as different questions may require different temporal perspectives, with some focusing on recent developments, while others may need a historical context. Aligning the date range with the research question ensures the SR remains relevant and precise. For transparency, it is important to report these restrictions, as they can introduce potential systematic bias.

### Step 3: evaluation of study selection and PRISMA flow diagram

The PRISMA flow diagram [4,11] is essential for tracking and reporting the study selection process. It outlines how studies were: 1) identified (including the number of records identified through database searching and other sources.), 2) screened (showing the number of records after duplicates are removed, number screened, and exclusions.), 3) selected (indicating the number of full-text articles assessed and reasons for exclusions at this stage.), and 4) included (the final number of studies incorporated into the systematic review and meta-analysis.) in the review. A properly constructed PRISMA flow diagram ensures transparency in study selection and is a critical element for readers to assess the rigor of the review; therefore, it should be included in the final manuscript.

It is encouraged to include the kappa statistic for inter-rater agreement during both study selection and data extraction. High kappa values indicate strong reliability between reviewers, while lower values may point to areas of potential bias or inconsistency. Additionally, it is beneficial to provide information on how any disagreements between reviewers were resolved, ideally through a structured consensus process, to enhance the transparency and rigor of the review.

To streamline the study selection process and lessen the effort required, several software tools, such as Covidence, Rayyan, DistillerSR, and EPPI-Reviewer, have been developed to assist researchers. Additionally, these tools can enhance both the efficiency and accuracy of the process. Each tool serves a specific purpose and has unique key features and strengths, as detailed in Table 2.

**Table 1.** Summary of the step-by-Step guide to appraising systematic reviews and meta-analyses.

Step	Description	Key tools/Guidelines
1. Research question and protocol	Ensure that the research question is clearly defined using the PICO framework (Population, Intervention, Comparison, Outcome). Check if the protocol was pre-registered (e.g. PROSPERO) to enhance transparency.	PROSPERO [9], PICO framework [10]
2. Literature search	Assess the comprehensiveness of the search strategy. Ensure databases like MEDLINE, Cochrane Library were searched, and search terms are provided. Justify any restrictions in language or publication date.	MEDLINE, Cochrane Library, PRISMA checklist [4,11]
3. Study selection and PRISMA Flow	Check if the study selection process is transparent and reproducible by reviewing the PRISMA flow diagram, which outlines how studies were identified, screened, and included. Verify whether any exclusions were justified.	PRISMA Flow Diagram [4,11], Covidence, Rayyan
4. Data extraction and study characteristics	Review the Table of Study Characteristics, ensuring it includes key details (study design, population, interventions, outcomes, risk of bias). Confirm that data extraction was standardized and consistent across studies.	Covidence, DistillerSR, Google Sheets, Excel
5. Risk of bias assessment	Evaluate the risk of bias in included studies. For RCTs, use Cochrane RoB 2; for non-randomized studies, use ROBINS-I. Ensure that all domains of bias (e.g. selection, performance, detection) are properly assessed.	Cochrane RoB 2, ROBINS-I For randomized controlled trials, apply the RoB 2 tool [12]. Older reviews may have used the JADAD scale [13], which assesses randomization, blinding, and withdrawals but lacks the depth and comprehensive bias assessment of RoB 2. For non-randomized studies, use the ROBINS-I tool [14]. In some older reviews, you may encounter the Newcastle-Ottawa Scale (NOS) [15]or its modified version [16], which assess selection, comparability, and outcomes but are less detailed compared to ROBINS-I.
6. Heterogeneity assessment	Assess heterogeneity using the I² statistic. High heterogeneity (I² > 50%) suggests the need for a random-effects model, while lower heterogeneity (I² ≤ 50%) might justify a fixed-effect model.	I² statistic, RevMan, CMA
7. Meta-analysis model choice	Evaluate whether the choice of a random-effects or fixed-effects model is appropriate based on the heterogeneity level. Ensure that the reasons for the model choice are clearly justified.	RevMan, Stata, CMA
8. Subgroup and sensitivity analysis	Check if appropriate subgroup or sensitivity analyses were performed to explore sources of heterogeneity or test the robustness of the findings.	Meta-regression, RevMan, Stata
9. Publication bias assessment	Examine funnel plots for potential publication bias. For studies with fewer than 10 studies, funnel plots may not be reliable. Additionally, use Egger’s test to statistically assess the symmetry of the funnel plot.	Funnel plots, Egger’s test
10. Statistical methods and effect estimates	Ensure the correct statistical methods were used for effect estimates (e.g. odds ratios, risk ratios, hazard ratios). Confirm that results are presented with 95% confidence intervals and visually displayed with forest plots.	RevMan, CMA, R (meta, metafor packages), Forest plots
11. Certainty of evidence	Use the GRADE framework to evaluate the overall certainty of the evidence, considering factors such as risk of bias, inconsistency, imprecision, publication bias, and indirectness. Indirectness refers to differences between the population, intervention, comparator, or outcomes in the included studies versus those most relevant to clinical practice.	GRADE framework [17]
12. Reporting and transparency	Confirm that the systematic review follows PRISMA guidelines and includes comprehensive reporting of results, data transparency (e.g. data availability on platforms like Open Science Framework), and acknowledgment of limitations.	PRISMA 2020 checklist, AMSTAR 2, Open Science Framework
13. Ethical considerations	Distinct ethical approval may not be required unless mandated by the policies of the authors’ institution or the regulations of their home country. However, conflicts of interest and funding sources must be clearly disclosed to maintain transparency.	Declaration of Conflicts of Interest
14. Conclusion and clinical relevance	Review whether the conclusions are justified by the results and whether the authors provide relevant implications for clinical practice, particularly in nephrology.	GRADE, Nephrology-Specific Outcomes (e.g. kidney function, proteinuria, dialysis adequacy)
15. Visual representation of studies	Bibliographic references should be directly linked to cited studies in tables and figures for ease of cross-referencing.	Chronological or reverse-chronological listing of studies is recommended, allowing reviewers and readers to better evaluate study sizes and temporal trends in results.
16. Providing constructive feedback	Offer positive feedback on well-executed areas first, then provide specific, actionable suggestions for improvement. Ensure that the tone is professional and constructive throughout the critique.	Professional feedback guidelines

**Table 2.** Examples of software tools for study selection process.

	Covidence	Rayyan	DistillerSR	EPPI-reviewer
Purpose	A software for offering a streamlined platform for screening abstracts, reviewing full texts, and resolving conflicts between reviewers.	A free web-based tool designed to assist in systematic reviews, focusing on speeding up the screening process.	A software that automates and organizes the literature screening process for systematic reviews.	A software for managing and analyzing data in systematic reviews, offering powerful tools for study selection, data extraction, and synthesis.
Key features	It allows for title/abstract screening, full-text review, data extraction, and the creation of PRISMA flow diagrams.	It allows for blind review of titles and abstracts, making it easier to reduce bias during the selection process.	It offers workflow management, automatic reference tracking, and flexible project customization.	It integrates with databases like PubMed and offers customizable workflows for study selection.
Strengths	User-friendly interface, enables multiple reviewers, and tracks conflicts for easier resolution.	User-friendly interface, enables multiple reviewers, and tracks conflicts for easier resolution.	Designed for large-scale systematic reviews and meta-analyses, supporting complex workflows and multiple users.	Its flexible approach to review structuring allows users to adapt to various review types.

### Step 4: evaluation of data extraction and table of study characteristics

Data extraction should be conducted using a predefined template that includes key demographic information, operational parameters, and outcomes established prior the review. A table of study characteristics is essential for organizing and presenting important details of included studies in a systematic review, as discussed below. Proper organization and clarity in this table enhance the transparency and reproducibility of the review. It is important for at least two review authors to perform data extraction to ensure inter-rater reliability, minimize the chances of data entry errors, and reduce the risk of bias from review authors.

For interventional studies, the data extraction process should include a description of the intervention, comparator, and study arms. However, for observational studies, the term ‘intervention’ should be replaced with ‘exposure’ to reflect the nature of the study design. This ensures consistency in reporting and distinguishes between controlled interventions and naturally occurring exposures.

During the data extraction phase, it is essential to document how missing data from primary studies were addressed. Reviewers should note whether the authors attempted to contact study authors for missing information or applied data imputation techniques to complete the dataset. Transparent reporting of these efforts helps ensure the completeness and reliability of the extracted data.

What to Include in a Table of Study Characteristics:

- Study Identifier: Include the authors, publication year, and study title. Arrange studies in chronological order whenever possible to enhance recognition of trends over time, allowing reviewers and readers, even those less familiar with the topic, to easily follow developments in study sizes, methodologies, and outcomes. This chronological presentation provides a clearer view of patterns and changes across the research timeline. Additionally, include a direct bibliographic identifier link (e.g., EndNote or other reference management software) for each study. This linking enables immediate access to reference details or original literature, supporting rapid cross-referencing with the study characteristics table.
- Study Design: Type of design (e.g., randomized controlled trial (RCT), cohort, case-control), setting, and country.
- Population Characteristics: Sample size, inclusion/exclusion criteria, and population details (e.g., age, gender, comorbidities).
- Intervention (or Exposure) and Comparator: For interventional studies, this refers to the description of the intervention and control groups. For observational studies, this should describe the exposure variable (e.g., high vs. low sodium intake, presence vs. absence of diabetes) and the comparison group (e.g., unexposed or different exposure level).
- Outcomes Assessment: Primary and secondary outcomes.
- Statistical Methods: Effect estimates (e.g., hazard ratios, odds ratios, risk ratios).
- Risk of Bias Assessment: Summary of bias evaluation using appropriate tools (e.g., Cochrane RoB 2 for RCTs).

This distinction between intervention and exposure is particularly relevant in nephrology research, where many studies focus on risk factors for chronic kidney disease (CKD) progression, dialysis outcomes, or transplant complications. For instance, a cohort study evaluating the impact of high dietary sodium intake on CKD progression would list sodium intake as the ‘exposure’ and patients with lower sodium intake as the ‘comparator.’ By making this modification, we ensure that systematic reviews maintain methodological precision when synthesizing evidence from both interventional and observational studies.

Once again, several software tools, such as Covidence, DistillerSR, and Excel or Google sheets, for data extraction are available to simplify this process. Researchers can either use the same software as for the selection phase or choose different software depending on their preferences.

### Step 5: quality assessment of included studies

The next crucial step in evaluating the SR/MA is to assess the quality of the included studies whether there are aspects that could introduce bias in the estimated effect, particularly the risk of overestimating or underestimating the true intervention effect. Proper interpretation and use of the quantitative results from an SR/MA must take these factors into account. Therefore, the SR/MA should provide a detailed risk of bias (RoB) assessment of individual studies based on their type of study designs.

There are a number of tools that the researchers may use to formally evaluate the RoB. Most tools are designed as scales, scoring various quality aspects to yield a summary score, or as checklists that include specific questions. For instance, older approaches like the Newcastle-Ottawa Scale (NOS) [15] and the JADAD scale [13] are commonly found in earlier SR/MA, particularly in the context of assessing the quality of non-RCTs and RCTs, respectively. Due to some limitations of these previous tools, the Cochrane Collaboration has recommended the ROBINS-I (Risk Of Bias In Non-randomized Studies of Interventions) [14] tool for non-RCTs and the RoB2 [9] tool, the updated version released in 2019, for RCTs when assessing quality.

The NOS [15] is used to evaluate cohort and case-control studies based on three main domains: selection of study groups, comparability of cohorts, and outcome assessment. While the NOS provides a quick and intuitive star-rating system, it has been criticized for its subjectivity and lack of specificity in some areas, which is why more recent tools such as ROBINS-I are preferred for modern meta-analyses. Additionally, the modified NOS enhances the original tool by refining its criteria for addressing study biases more comprehensively. This version may be more applicable for nephrology-specific studies, where complex interventions or patient populations warrant a more nuanced assessment of risk factors and confounding variables. For RCTs, the JADAD scale was one of the earliest tools used to assess methodological quality, focusing on randomization, blinding, and the handling of withdrawals. Although it remains widely cited, its limitations, such as overlooking allocation concealment, have led to its replacement by more rigorous tools like RoB-2 in current systematic reviews.

The QUADAS-2 (Quality Assessment of Diagnostic Accuracy Studies) tool [23] is the gold standard for evaluating the risk of bias in DTA meta-analyses. This tool assesses four key domains: patient selection, index test performance, reference standard, and flow/timing of the study. In nephrology, an important consideration is verification bias, where the reference standard (e.g. kidney biopsy) is not applied to all patients, leading to an overestimation of test accuracy. Additionally, spectrum bias must be considered, as test performance may vary between high-risk (hospitalized) and low-risk (outpatient) populations.

### Step 6: evaluation of data synthesis and meta-analysis

A significant step in conducting a systematic review is the thoughtful evaluation of whether it is appropriate to combine the numerical results from all or some of the included studies. This process, known as meta-analysis, synthesizes quantitative data across various studies to yield a pooled effect estimate along with its confidence interval. In clinical trials, different statistical models can be utilized depending on the type of outcome data (e.g. dichotomous, continuous, ordinal, rate, or time-to-event) and the study design, resulting in different measures of effect estimates (Table 3). For example, dichotomous outcomes can be compared between two groups using risk ratios (RRs), odds ratios (ORs), or risk differences (RDs), while continuous outcomes can be compared using mean differences or standardized mean differences. Furthermore, it is important to consistently report effect estimates along with 95% confidence intervals (CIs) to demonstrate the precision of the effect estimates. Narrow CIs indicate greater precision, while wide CIs imply less certainty in the findings.

**Table 3.** Summary of commonly reported effect measures based on data type.

Type of data	Effect measure
Dichotomous	Odds Ratio (OR), Risk Ratio (RR), Risk Difference (RD)
Continuous	Mean Difference (MD), Standardized Mean Difference (SMD)
Ordinal	Proportional Odds Ratio
Count or rate	Rate Ratio, Rate Difference, Incidence Rate Ratio
Time-to-event	Hazard Ratio (HR)
Prevalence/Incidence	Pooled Prevalence, Incidence Rate
Predictive accuracy	Area Under the Curve (AUC)
Diagnostic test accuracy	Sensitivity, Specificity, Positive Predictive Value (PPV), Negative Predictive Value (NPV), Diagnostic Odds Ratio (DOR), Summary Receiver Operating Characteristic (SROC) Curve, Likelihood Ratios (LR+ / LR-)

Abbreviations: AUC: Area Under the Curve; HR: Hazard Ratio; IRR: Incidence Rate Ratio; MD: Mean Difference; OR: Odds Ratio; PP: Pooled Prevalence; RD: Risk Difference; RR: Risk Ratio; SMD: Standardized Mean Difference.

Unlike intervention meta-analyses that commonly use fixed- or random-effects models, DTA meta-analyses require specialized statistical approaches. The hierarchical summary receiver operating characteristic (HSROC) model is recommended for pooling sensitivity and specificity, as it accounts for study-level heterogeneity in diagnostic performance. Additionally, bivariate models can be used to estimate joint distributions of sensitivity and specificity, improving interpretability for clinical application. These approaches are particularly valuable in nephrology, where tests such as urine albumin-to-creatinine ratio (UACR) and serum FGF-23 levels exhibit threshold-dependent accuracy.

In a SR/MA of comparative studies, it is usual to present summary statistics using forest plots, which visually represent individual study estimates and the overall pooled effect size. Each horizontal line in a Forest plot represents a study’s effect estimate along with its 95% CI, while the vertical line denotes the line of no effect (e.g. an OR or RR of 1). The pooled effect is shown as a diamond, with the center representing the estimate and the width indicating the 95% CI. Additionally, forest plots are essential for showing the consistency or variability of results across studies, particularly in meta-analyses of treatment effects (e.g. RRs, ORs, HRs).

Another important factor that should be taken into consideration is variability between studies, referred to as heterogeneity. Heterogeneity can result from differences in one or more PICO components, such as the clinical characteristics of the populations involved, the types of intervention utilized across studies, or differences in methodology, including outcome measures and study designs.

The usual way of assessing whether there is true heterogeneity in a meta-analysis has been to use the Q test, a statistical test defined by Cochran [24,25]. The Q test is computed by summing the squared deviations of each study’s effect estimate from the overall effect estimate, weighting the contribution of each study by its inverse variance. Under the hypothesis of homogeneity among the effect sizes, the Q statistic follows a chi-square distribution with k − 1 degrees of freedom, where k represents the number of studies. Not rejecting the homogeneity hypothesis assumed that the estimated effect sizes only differ by sampling error (indicating no significant heterogeneity) [26]. In contrast, rejecting the homogeneity assumption suggests that a statistically significant result for heterogeneity may indicate an issue with variability either within studies, between studies, or both. However, interpreting this test requires caution, as it has low power in meta-analyses with small sample sizes or few studies. A non-significant result should not be taken as evidence of no heterogeneity. For this reason, a P value of 0.10 is sometimes used instead of the usual 0.05 to determine statistical significance. Additionally, the Q statistic only indicates the statistical significance of heterogeneity, not its actual extent [27].

Another approach to quantifying true heterogeneity in a meta-analysis is by estimating the between-studies variance (τ^2^). In a random-effects model, τ^2^ reflects how much the true population effect sizes differ across studies. However, τ^2^ values cannot be compared across meta-analyses that use different effect-size indices (e.g. correlation coefficients, standardized mean differences), as τ^2^ depends on the specific effect metric used. In order to overcome the shortcomings of the Q test and the τ^2^, the I^2^ statistics [28] has been proposed for assessing heterogeneity in a meta-analysis. The I^2^ statistic [28,29], on the other hand, offers a measure of variability, with 0% indicating that any variability is solely attributed to chance. As I^2^ values increase, they reflect higher levels of unexplained variability. An I^2^ value of ≤ 25% suggests low heterogeneity, 26–50% indicates moderate heterogeneity, and values greater than 50% signify high heterogeneity [26]. For complete transparency, including the I^2^ statistic in the abstract is essential for clearly expressing the level of heterogeneity in the meta-analysis. A well-conducted SR/MA should address heterogeneity, explain how this guided the researchers’ decisions on data handling, and explore potential sources of it as follows:

Heterogeneity is particularly relevant in nephrology due to diverse patient populations, differences in dialysis modalities (e.g. hemodialysis vs. peritoneal dialysis), and variations in study endpoints (e.g. progression to ESKD, changes in estimated glomerular filtration rate [eGFR], or cardiovascular mortality). For example, in a meta-analysis comparing renin-angiotensin-aldosterone system (RAAS) inhibitors in CKD patients, high heterogeneity may arise if studies include different CKD stages, comorbid conditions, or definitions of progression. Subgroup analyses stratified by baseline eGFR, proteinuria levels, or dialysis status can help identify sources of heterogeneity and refine clinical interpretation.

In DTA meta-analyses, heterogeneity is often driven by differences in patient populations (e.g. CKD vs. AKI), study designs, and diagnostic thresholds. For instance, studies assessing urinary NGAL for AKI detection may use different cutoff values, leading to variability in reported sensitivity and specificity. When heterogeneity is present, hierarchical models such as the bivariate random-effects model or the hierarchical summary receiver operating characteristic (HSROC) model should be employed. These models account for the correlation between sensitivity and specificity and provide a more accurate pooled estimate.

#### Data handling with the appropriate model choice

Generally, the choice of model is not rigid and does not depend solely on the I^2^ value, but depends on the circumstances and the researcher’s interpretation of the model’s underlying assumptions. In the presence of heterogeneity, a random-effects meta-analysis weights studies more equally than a fixed-effect model. It is usually the preferred choice because it accounts for variability both within and between studies. The random-effects model seeks to generalize the findings beyond the included studies by assuming that the selected studies are random samples from a larger population. Additionally, it assumes that the true effects differ between studies, such as due to variations in populations, interventions, or methodologies. In comparison, fixed-effect meta-analysis assumes that all studies share a common treatment effect, and any differences in effect sizes are due to sampling error. The summary treatment effect is calculated as a weighted average of the individual study effects, with studies that have more precise results (lower variance) receiving greater weight. Therefore, the fixed-effect model is appropriate when there are too few studies to reliably estimate the variance between studies or when larger studies are more methodologically robust or more accurately represent the typical use of the intervention in practice. This model will provide a greater weight to the larger, better-quality study.

#### Explore sources of heterogeneity

Nephrological studies often involve diverse patient groups and treatments. Therefore, systematic reviews should thoroughly evaluate all potential variables and sources of heterogeneity. In nephrology, heterogeneity can arise from variations in patient demographics (e.g. age, comorbidities, and kidney function), intervention types (e.g. dialysis modalities, pharmacologic therapies), and outcome measures (e.g. GFR decline, proteinuria, and mortality). Addressing these sources of heterogeneity requires a comprehensive and structured approach. To investigate the sources of heterogeneity in meta-analysis is to identify study-level characteristics, such as study quality, differences in study populations, or variations in interventions, that contribute to the variation in study results. The commonly used methods for doing this are subgroup analysis and meta-regression. Ideally, this section should be clearly defined in the review protocol. Trustworthy conclusions can only be derived from analyses that are truly pre-specified before inspecting the results of the studies. Another common approach is to conduct sensitivity analyses, in which one or more studies are excluded at a time to assess the effect of removing each study or set of studies on the overall results and the heterogeneity between studies. Identifying ‘outlier’ studies can also provide insights into the potential reasons for the observed between-study heterogeneity. However, unplanned, *post hoc* exclusions are particularly discouraged because they can introduce biases. Authors should carefully design sensitivity analyses during the protocol stage to ensure the robustness and credibility of their meta-analytic conclusions.

Sensitivity analyses are critical for evaluating the robustness of systematic reviews, particularly in nephrology, where patient populations and interventions are often complex. One relevant approach is stratified sensitivity analysis, which examines subgroups based on factors such as dialysis modality (e.g. hemodialysis vs. peritoneal dialysis) or baseline kidney function (e.g. CKD stages). Another useful method is excluding studies with high risk of bias to determine their influence on overall findings. For example, in a meta-analysis assessing the efficacy of renin-angiotensin-aldosterone system inhibitors in CKD patients, sensitivity analysis might exclude trials with inadequate blinding to assess whether their inclusion skews results. By incorporating these analyses during the protocol design phase, authors can ensure that systematic reviews provide nuanced insights while maintaining methodological rigor. Providing clear examples of sensitivity analyses enhances the practical relevance of the review and supports evidence-based decision-making in nephrology.

Publication bias is an important limitation to consider in systematic reviews. One common method to assess this bias is through funnel plots, which visually compare study size (or standard error) against effect size. Ideally, when publication bias is minimal, studies should be symmetrically distributed around the pooled effect estimate, forming an inverted funnel shape. However, asymmetry in the funnel plot may indicate potential bias, such as the underreporting of smaller studies with null or negative results. It is important to note that funnel plots are generally unreliable when fewer than 10 studies are included, as their ability to detect asymmetry is significantly reduced in small sample sizes. Egger’s regression test can supplement funnel plots by statistically assessing asymmetry [30]). A p-value below 0.05 in Egger’s test suggests possible publication bias. However, similar to funnel plots, Egger’s test is also less reliable when fewer than 10 studies are included in the meta-analysis.

In diagnostic test accuracy (DTA) meta-analyses, publication bias may present differently, often through selective reporting of high-sensitivity or high-specificity thresholds, particularly when multiple cutoffs are examined in primary studies [31]. Unlike traditional funnel plots used in intervention meta-analyses, DTA studies require alternative methods such as Deeks’ funnel plot, which plots the diagnostic odds ratio (DOR) against the inverse of the study size standard error. If Deeks’ plot exhibits asymmetry, this suggests potential bias, necessitating further sensitivity analyses [31].

Publication bias is a notable concern in nephrology research, especially for topics with industry-sponsored trials, such as novel phosphate binders or sodium-glucose co-transporter-2 (SGLT2) inhibitors for chronic kidney disease (CKD). For instance, a meta-analysis evaluating the effects of SGLT2 inhibitors on CKD progression may be affected by publication bias if smaller studies with neutral or negative findings are underrepresented. In such cases, funnel plots and Egger’s test should be interpreted in the context of clinical relevance, and additional efforts should be made to incorporate unpublished or grey literature, including conference abstracts from ASN Kidney Week or the ERA Congress.

Several programs and software tools are available to facilitate this process as detailed in Table 4.

**Table 4.** Examples of programs and software tools for data synthesis and meta-analysis.

	Review manager (RevMan)	Comprehensive meta-analysis (CMA)	Stata	R (meta, metafor, and metaSEM packages)	MetaXL
Purpose	RevMan is widely used for conducting meta-analyses, developed by the Cochrane Collaboration,	CMA is a standalone program designed for performing meta-analyses of various effect measures.	Stata is a general statistical software that includes meta-analysis commands for synthesizing data.	R is a programming language used for statistical computing and includes several packages specifically designed for meta-analysis (e.g. meta, metafor).	MetaXL is an Excel-based add-in designed for conducting meta-analyses.
Key features	Includes tools for creating forest plots, calculating effect estimates, and conducting subgroup analyses.	Supports different effect sizes (odds ratios, risk ratios, hazard ratios) and provides advanced statistical tools for sensitivity analyses and meta-regression.	Allows for complex meta-analytic models, including random-effects, fixed-effects, and meta-regression.	R packages allow for complex meta-analysis, including meta-regression and advanced statistical tests.	Simple interface for conducting fixed-effect and random-effects meta-analyses, forest plots, and publication bias assessments.
Strengths	User-friendly, ideal for Cochrane systematic reviews, and includes built-in risk of bias assessment tools.	Flexible, powerful, and easy to handle for large datasets.	High-level programming options and robust data handling.	Highly flexible, open-source, and supports custom analysis scripts.	Easy to use for those familiar with Excel but lacks the depth of dedicated statistical programs like RevMan or CMA.

### Step 7: results presentation

To enhance clarity for readers and reviewers regarding the results of SR/MA, we suggest incorporating the figures and tables specified in Table 5 for the presentation of results.

**Table 5.** Overview of figures and tables to include in results Presentation.

Part of manuscript	Figures	Tables
Main	PRISMA flow diagram for the search strategy process Forest plot for primary outcome Meta-regression analysis graph, if applicable	Study characteristics of included articles
	Subgroup analysis of primary outcome	
Supplementary	Forest plot for secondary outcome Funnel plot and Egger’s test*	Search strategy PRISMA or AMSTAR checklist
	Subgroup analysis of secondary outcomes Risk of bias summary*, if applicable	

*This can alternatively be included as a main figure/table.

#### Evaluation certainty of evidence

To thoroughly clarify the presentation of results in the SR/MA, the Grading of Recommendations Assessment, Development, and Evaluation (GRADE) [17] is a widely used framework that helps determine the overall certainty of evidence and the strength of recommendations by categorizing evidence quality into four levels: high, moderate, low, or very low. Even if systematic review authors do not explicitly apply GRADE, aspects such as study bias, result inconsistency, evidence indirectness, imprecision, and reporting bias should still influence the appraiser’s confidence in the outcome measures.

The GRADE framework assesses the certainty of evidence based on five key domains: risk of bias, inconsistency, imprecision, publication bias, and indirectness. Indirectness is particularly important in nephrology, where studies often use surrogate markers such as albuminuria, creatinine clearance, or composite endpoints instead of hard clinical outcomes like end-stage kidney disease (ESKD) or mortality. For example, a meta-analysis evaluating the renoprotective effects of sodium-glucose co-transporter-2 (SGLT2) inhibitors may report improvements in proteinuria and eGFR slope, but these surrogate endpoints may not fully capture long-term benefits on kidney survival or cardiovascular outcomes.

Additionally, indirectness can arise when study populations differ from real-world nephrology patients. For instance, a systematic review of immunosuppressive therapies for lupus nephritis may predominantly include clinical trial participants with mild disease activity, limiting generalizability to patients with severe nephrotic syndrome or those receiving concomitant therapies. Recognizing and addressing indirectness ensures that systematic reviews and meta-analyses provide accurate and clinically relevant conclusions that can inform nephrology practice.

When assessing the certainty of evidence in nephrology systematic reviews, specific considerations include the risk of bias in long-term follow-up studies, indirectness in surrogate outcomes (e.g. albuminuria vs. hard endpoints like ESKD), and imprecision in rare kidney diseases. For instance, in a systematic review evaluating immunosuppressive therapies for lupus nephritis, the GRADE approach should consider whether surrogate markers (e.g. proteinuria reduction) accurately reflect long-term renal survival. Additionally, given the reliance on observational studies for rare conditions like atypical hemolytic uremic syndrome (aHUS), rating up the certainty of evidence for large, well-conducted cohort studies may be warranted.

### Step 8: discussion and conclusions

It is crucial to acknowledge the potential limitations of systematic reviews in the discussion section, which should be considered by both researchers and reviewers. These may include the quality of the included studies, such as selection bias, inadequate blinding, attrition bias, and selective outcome reporting. Other issues to consider in each included study are small sample sizes, short follow-up periods, and notable differences in baseline characteristics between comparison groups. Additionally, any inconsistencies, such as significant heterogeneity and imprecision that could lead to Type I and Type II errors, should be mentioned if they exist.

In the conclusion section, it should clearly outline the main findings related to the review question and summarize their clinical significance for implications in clinical practice and/or policy, while also stating the level of evidence. Furthermore, it should clearly offer the implications for research by summarizing the uncertainties of the existing evidence base, highlighting knowledge gaps, and emphasizing the need for further primary research.

By adopting a structured approach to critical appraisal, nephrologists can ensure that systematic reviews and meta-analyses provide meaningful insights into kidney disease management. Given the rapid evolution of nephrology research, including advances in precision medicine, artificial intelligence, and nephroprotective therapies, it is crucial to critically evaluate the applicability of pooled evidence to real-world clinical practice. This guide equips nephrologists with the necessary tools to assess the strengths and limitations of systematic reviews, ultimately enhancing patient care through more informed clinical decision-making.

### Step 9: nephrology-specific considerations

Recently, several clinical trials in nephrology have utilized composite endpoints that include clinical events such as the onset of end-stage kidney disease (ESKD), the initiation of kidney replacement therapy (KRT), receiving a kidney transplant, and death due to kidney failure, along with a sustained decrease in GFR of varying percentage cutoffs (e.g. 30%, 40%, 50%, and 57%). Depending on the trial setting, different components may be included or omitted. It is important to examine the specifics of reported outcomes in each study before combining them in a meta-analysis. Some researchers consider hierarchical composite endpoints (HCEs) to rank components based on their clinical significance and separate them prior to analyzing the data in meta-analysis [32].

Moreover, outcomes reported in nephrology trials often include patient-reported outcomes (PROs), such as fatigue, uremic pruritus, pain, and quality of life. However, incorporating PROs can raise concerns regarding their validity (trustworthiness of the methods) and interpretability (meaning of the results), leading to uncertainty among many authors of systematic reviews. The definition of a specific PRO may vary between studies, which may necessitate the use of different measurement instruments. Even when definitions are similar, authors might select different tools to evaluate it. For example, the following measurements are all validated patient-reported pruritus severity score that an investigator may use in a primary study to assess an intervention’s effectiveness for treating uremic pruritus: the visual analog scale (VAS), the numeric rating scale (NRS), the skindex-10, and the 5-D questionnaire. Systematic reviewers need to determine how to classify these PROs and when to combine the results. To ensure suitable pooling of data from two related PROs, it is essential to: 1) have strong longitudinal correlations in individual patient data showing changes in both measures, along with evidence that the interventions demonstrate similar responsiveness, and 2) establish correlations of the differences between before and after measurements between groups, across studies. If such data cannot be found, combining the results may not be appropriate.

### Step 10: ethical considerations

The core purpose for incorporating ethics into the checklist of systematic reviews is to raise awareness in the scientific community about the importance of maintaining high ethical standards in research on humans. This proposal would also encourage reviewers to pinpoint studies that were unacceptably unethical, which may raise moral concerns about the use of their results. Concerns regarding ethical quality significantly intersect with the fundamental issues of validity, reliability, and generalizability of research findings. As such, it is essential to include ethical assessment in systematic reviews for both prudential and moral considerations. In addition, review authors should take into account the funding sources and potential conflicts of interest of the study, as these factors can influence the evaluation of the directness and heterogeneity of study results, as well as the assessment of bias risk within the studies, and in the syntheses due to missing results.

### Step 11: providing constructive feedback

As a peer reviewer, it is essential to provide comprehensive and transparent feedback to authors, ideally supported by the best available evidence. Starting the feedback by highlighting positive aspects of the manuscript can be beneficial, as there is little advantage in focusing solely on negative points. Typically, authors dedicate significant time and effort to their work, so acknowledging their contributions is important. The next section of the peer review report should address any major issues identified, including any critical flaws. It can be helpful to provide suggestions and frame these concerns as questions for the authors, as they may have overlooked them or not considered the relevant clinical issues or methods raised by the reviewer. Additionally, effective peer reviewers should stay well-informed about recent advancements in the literature within their area of expertise to ensure thoroughness. Systematic review reports may present with several inherent issues that lead peer reviewers to request significant modifications. It is crucial for critiques to maintain a professional tone, and reviewers should avoid suggesting that authors conduct an entirely new systematic review when addressing these issues. Lastly, when invited to peer review a systematic review protocol, reviewers may utilize the PRISMA-P checklist [33] to streamline the process and avoid potential pitfalls in key aspects of the review.

### Step 12: common biases to look for

Review authors have to recognize and identify the common biases that may be present in the included studies of the systematic review by addressing specific questions in each relevant domains (Table 6).

**Table 6.** Summary of common types of biases and their relevant domains for review authors’ assessment.

Type of bias	Description	Relevant domains	Nephrology-specific considerations
Selection bias	Systematic differences between baseline characteristics of study groups.	Sequence generation, allocation concealment.	Nephrology trials often face selection bias due to differential inclusion of patients based on kidney function (e.g. eGFR thresholds), dialysis status, or transplantation status, leading to non-representative samples.
Performance bias	Systematic differences in care or exposure to other factors between groups.	Blinding of participants, personnel, and outcome assessors.	In interventional studies, nephrology patients often receive concomitant therapies (e.g. RAAS inhibitors, diuretics) that may confound treatment effects. Blinding may be compromised in dialysis-related trials due to differences in treatment administration (e.g. hemodialysis vs. peritoneal dialysis).
Attrition bias	Systematic differences in withdrawals between groups.	Incomplete outcome data.	High attrition rates are common in CKD studies due to disease progression, kidney transplantation, or mortality, leading to potential loss-to-follow-up bias. Intention-to-treat analyses should be emphasized in these studies.
Detection bias	Systematic differences between groups in how outcomes are determined.	Blinding of participants, personnel, and outcome assessors.	Kidney function markers (e.g. eGFR, proteinuria) are prone to variability due to hydration status, acute illness, and laboratory measurement differences, potentially influencing outcome assessment in observational and interventional studies.
Reporting bias	Preferential reporting of favorable results within a study.	Selective outcome reporting.	In nephrology, composite endpoints (e.g. time to ESKD, mortality, and significant GFR decline) may lead to selective reporting of individual components if they yield significant results, potentially distorting interpretation.
Survivorship bias	Exclusion of patients who do not survive long enough to be included in studies.	Selection criteria.	Particularly relevant in dialysis and kidney transplantation studies, where only patients who survive long-term follow-up are included, potentially overestimating treatment benefits in healthier cohorts.
Confounding by indication	Bias introduced when treatment is influenced by disease severity.	Study design, confounding adjustment.	Nephrology patients with advanced CKD may preferentially receive intensive interventions (e.g. SGLT2 inhibitors, dialysis initiation), making it challenging to isolate treatment effects from disease severity-related outcomes. Propensity score matching and instrumental variable analysis can help mitigate this bias.
Center effect bias	Variability in outcomes based on differences between study centers.	Study generalizability, external validity.	Multicenter nephrology trials may show significant variability due to differences in dialysis protocols, transplant practices, or CKD management strategies across institutions. Sensitivity analyses accounting for center-specific effects are recommended.

**Figure 1.**
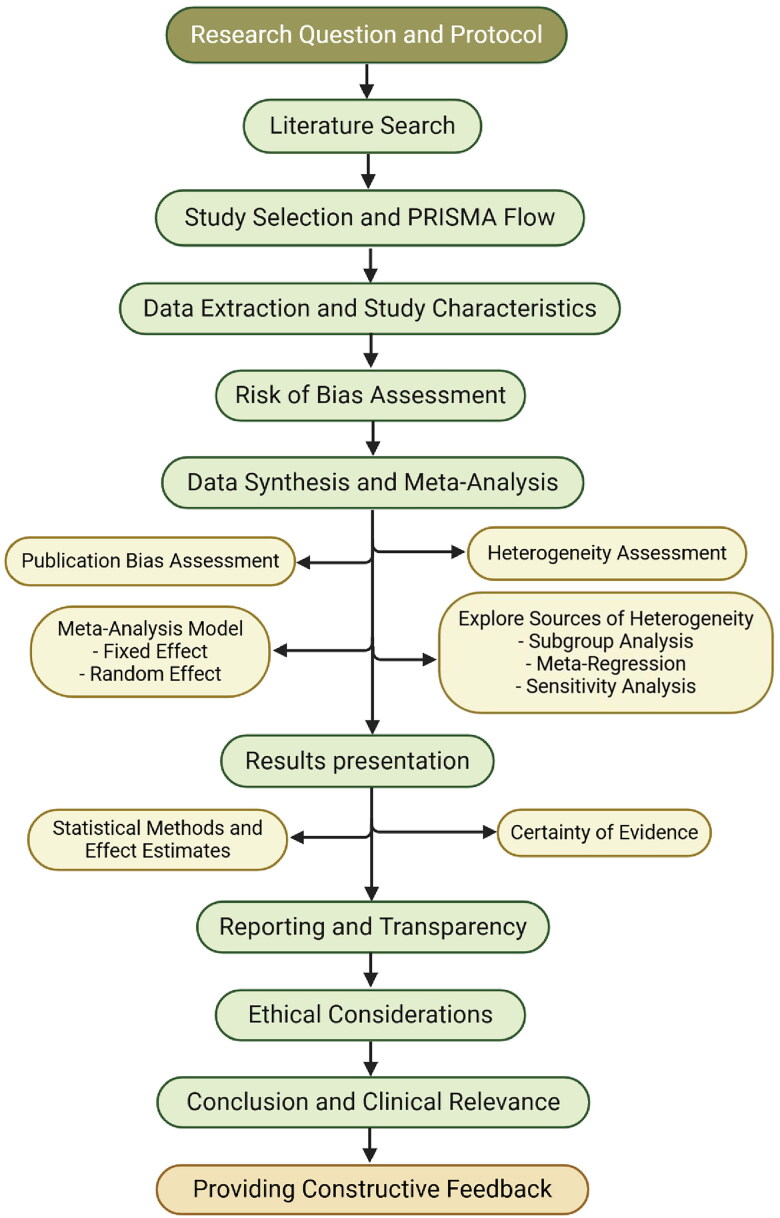
Flow diagram checklists for appraising systematic reviews and meta-analyses. Created by BioRender.com.

In summary, the critical appraisal of systematic reviews and meta-analyses in nephrology requires a comprehensive and structured approach. This process encompasses several key steps (Figure 1): ensuring compliance with PRISMA 2020 guidelines; utilizing the AMSTAR 2 tool for methodological assessment; applying appropriate risk of bias tools such as Cochrane RoB 2 and ROBINS-I; evaluating evidence certainty through the GRADE framework; and verifying the use of suitable meta-analysis techniques, including model selection, meta-regression, and sensitivity analyses. Moreover, it is crucial to examine data transparency and reporting quality. Best practices in this field also include pre-registration of review protocols, clear acknowledgment of study limitations, and thorough reporting of sensitivity and subgroup analyses along with detailed statistical information.

### Step 13: miscellaneous considerations

Some authors choose to include grey literature—publications that have not undergone formal peer review—in their systematic reviews. While grey literature is not always part of the review process, it may surface during review. Grey literature can encompass sources such as reports, dissertations, conference abstracts, and government documents. Although these sources may broaden the evidence base, reviewers should be aware of their variable quality and lack of formal peer-review processes, which could impact the strength and reliability of findings.

Recently, the publication of umbrella reviews has notably increased in medical research, demonstrating their growing significance in scientific literature. However, this rising trend also brings several concerns about the methodology and interpretation of such comprehensive research syntheses. This kind of research is dependent on existing meta-analyses, which can lead to potential biases, missing studies, and methodological challenges inherited from the original research. Issues of heterogeneity in exposure and outcome assessment persist, and the approach is constrained by only including previously synthesized research, thus potentially missing the most recent evidence. Additionally, despite available standardized methods, many authors do not adhere to established guidelines, which introduces uncertainty into the knowledge synthesis. The fundamental solution proposed is to focus on encouraging high-quality primary research to improve the reliability of both primary and secondary studies.

The last point to mention is that diagnosis plays a crucial role in clinical treatment, and meta-analysis of diagnostic tests can provide more statistically robust and precise results when multiple small studies are available on the same test and patient population. Despite the potential benefits, there are significant challenges in conducting these analyses. Many researchers make errors in their approach, often incorrectly defaulting to SMD calculations instead of properly creating summary receiver operating characteristic (SROC) curves and sensitivity/specificity plots, which are more appropriate for evaluating diagnostic test accuracy. Review authors are reminded that choosing inappropriate meta-analytic methods can result in findings that do not offer useful practical insights for clinical practice.

### Step 14: expectations during revision

Most journals provide specific guidelines on how to track revisions made during the peer review process. For example, *Renal Failure* requests that authors use yellow highlighting to denote interim changes made in response to reviewers’ comments, allowing peer reviewers to quickly identify modifications. Furthermore, authors are expected to submit a detailed rebuttal letter, addressing each reviewer’s point or concern individually, with a clear explanation of how each has been resolved in the revised manuscript. This approach respects the significant time and effort that peer reviewers volunteer in assessing manuscripts and ensures that their insights are thoughtfully incorporated.

## Conclusion

Critical appraisal of systematic reviews and meta-analyses is a crucial skill for nephrologists engaged in peer review. By systematically evaluating the key components, applying the recommended tools and guidelines, and considering best practices in meta-analysis techniques, we can offer valuable feedback that enhances the quality of evidence in nephrology. It is essential to always consider the clinical relevance of findings within the nephrology context when reviewing these studies. By adhering to these guidelines and best practices, we can ensure a thorough and effective critical appraisal process that maintains the integrity and quality of published research in nephrology, ultimately contributing to improved patient care through evidence-based practice.

## Data Availability

All data that support this study has been provided and are also available on request from the corresponding author.
